# Variability and Global Distribution of Subgenotypes of Bovine Viral Diarrhea Virus

**DOI:** 10.3390/v9060128

**Published:** 2017-05-26

**Authors:** Kadir Yeşilbağ, Gizem Alpay, Paul Becher

**Affiliations:** 1 Department of Virology, Faculty of Veterinary Medicine, Uludag University, TR-16059 Bursa, Turkey; gizemalpay@uludag.edu.tr; 2 Institute for Virology, Department of Infectious Diseases, University of Veterinary Medicine, D-30559 Hannover, Germany

**Keywords:** bovine viral diarrhea virus, epidemiology, global distribution, genetic diversity, subgenotyping

## Abstract

Bovine viral diarrhea virus (BVDV) is a globally-distributed agent responsible for numerous clinical syndromes that lead to major economic losses. Two species, BVDV-1 and BVDV-2, discriminated on the basis of genetic and antigenic differences, are classified in the genus *Pestivirus* within the *Flaviviridae* family and distributed on all of the continents. BVDV-1 can be segregated into at least twenty-one subgenotypes (1a–1u), while four subgenotypes have been described for BVDV-2 (2a–2d). With respect to published sequences, the number of virus isolates described for BVDV-1 (88.2%) is considerably higher than for BVDV-2 (11.8%). The most frequently-reported BVDV-1 subgenotype are 1b, followed by 1a and 1c. The highest number of various BVDV subgenotypes has been documented in European countries, indicating greater genetic diversity of the virus on this continent. Current segregation of BVDV field isolates and the designation of subgenotypes are not harmonized. While the species BVDV-1 and BVDV-2 can be clearly differentiated independently from the portion of the genome being compared, analysis of different genomic regions can result in inconsistent assignment of some BVDV isolates to defined subgenotypes. To avoid non-conformities the authors recommend the development of a harmonized system for subdivision of BVDV isolates into defined subgenotypes.

## 1. Introduction

Bovine viral diarrhea virus (BVDV) is an important pathogen of cattle with a global distribution and causes major economic losses [1]. The two species BVDV-1 and BVDV-2 are members of the *Pestivirus* genus within the family *Flaviviridae*. Currently, the International Committee on Taxonomy of Viruses (ICTV) recognizes four approved *Pestivirus* species: *BVDV-1*, *BVDV-2*, *Classical swine fever virus* (CSFV), and *Border disease virus* (BDV) [2]. Moreover, a growing number of additional tentative *Pestivirus* species from various domestic and wildlife animal species has been described: (i) “Giraffe” virus, comprising an isolate obtained from a giraffe in Kenya, that caused mucosal disease-like symptoms, as well as one bovine isolate [3,4]; (ii) “Pronghorn” virus, isolated from a blind pronghorn antelope in the USA [5]; (iii) “Bungowannah” virus, that was isolated from pigs in Australia [6]; and (iv) atypical “HoBi-like” pestiviruses detected in the serum and other samples from bovine and buffalo [7,8,9]. Recently, additional putative new pestivirus species have been described, including Aydin-like viruses isolated from sheep and goats in Turkey [10], atypical porcine pestivirus causing congenital tremor in piglets [11,12], a pestivirus from a bat [13], and a pestivirus from rats [14]. In contrast to the approval and classification of *Pestivirus* species, subdivision of BVDV-1 and BVDV-2 into genetic groups is not an issue of the ICTV, but widely used in studies characterizing BVDV isolates. 

Although pestiviruses were initially designated according to their host of origin, infections with BVDV have been detected in diverse domestic and wildlife animal species, including cattle, sheep, goat, pig, deer, buffalo, bison, and alpaca [15,16,17]. In addition to its respiratory, gastroenteric and reproductive clinical consequences, intrauterine infection of the fetus with BVDV can result in the birth of immunotolerant, persistently infected (PI) animals. These PI animals shed large amounts of virus during their life and are the main source of virus transmission to susceptible animals. Thus, identification and elimination of PI animals, together with the implementation of biosecurity measures, are crucial for control and prevention of the disease. Vaccination can represent an accompanying tool to prevent BVDV, but without removing PI animals it does not enable the elimination of the virus in a susceptible population. The genetic variations described for BVDV-1 and BVDV-2 may be implicated in disease control as diagnostics and vaccines that work well against homologous strains can be less efficacious for genetically-distinct viruses [18,19,20,21].

## 2. Genomic Organization

The genome of BVDV consists of a positive-stranded RNA molecule approximately 12.3 kb in length [22]. For most cytopathogenic BVDV strains considerably larger genomes have been described [23]. The single open reading frame (ORF) of the BVDV genome is flanked by untranslated regions (UTRs). The ORF encodes one large polyprotein, which is processed by cellular and viral proteases into four structural, and eight nonstructural, proteins ([24] and references therein). These mature proteins are N^pro^, C, E^rns^, E1, E2, p7, NS2, NS3, NS4A, NS4B, NS5A, and NS5B. The first protein in the polyprotein, N^pro^, is a nonstructural viral autoprotease producing its own C-terminus. The nucleocapsid protein C and the three envelope glycoproteins E^rns^, E1, and E2 represent the structural proteins of BVDV. E2 is a highly-variable, immunologically-dominant glycoprotein in pestiviruses and the main target of neutralizing antibodies. The remaining mature proteins are nonstructural. The 5′UTR is a highly-conserved part of the viral genome and comprises an internal ribosome entry site essentially implicated in the translation of the viral polyprotein. The characteristics and functions of the individual pestivirus proteins, properties of the 5′ and 3′UTR, as well as molecular aspects of viral replication and cytopathogenicity of BVDV have been recently reviewed [23,24].

## 3. Mechanisms of Genetic Changes in BVDV Genomes

Genetic changes in pestivirus genomes result from three different processes: (1) accumulation of point mutations resulting from the error-prone nature of the viral RNA-dependent RNA polymerase; (2) non-homologous RNA recombination; and (3) homologous RNA recombination. Assuming that the mutation rate of pestiviruses is similar to those reported for other RNA viruses, it can be roughly estimated that one point mutation is introduced into the pestivirus genome per replication cycle [25,26]. For BVDV-1, different evolutionary rates have been published. Analysis of 5′UTR sequences of BVDV revealed a mean evolutionary rate of 9.3 × 10^−3^ substitutions/site/year for the investigated sequences [27]. Moreover, evolutionary rates of 5.9 × 10^−4^ and 1.26 × 10^−3^ substitutions/site/year have been reported for the 5′UTR and E1–E2 regions, respectively [28]. In addition, non-homologous RNA recombination can lead to the generation of cytopathogenic (cp) BVDV variants and a large variety of different genomic alterations have been described for cp pestiviruses ([23] and references therein). The emergence of cp BVDV in persistently-infected animals is crucial for the induction of fatal mucosal disease. Accordingly, RNA recombination, the emergence of cp BVDV, and pathogenesis of lethal mucosal disease are closely-linked processes [23]. Furthermore, homologous RNA recombination in pestivirus populations including BVDV-1 and BVDV-2 has been described [29,30,31]. Analysis of 125 complete pestivirus sequences provided evidence that the genomes of two BVDV-1, one BVDV-2, and four CSFV strains evolved from homologous recombination [30]. Depending on the genomic region used for phylogenetic analysis, the two recombinant BVDV-1 strains, ILLNC and 3156, are classified as either BVDV-1a or BVDV-1b, while the genome of the BVDV-2 strain JZ05-1 resulted from a recombination event between BVDV-2a and BVDV-2b. A recent in silico study on the evolution of BVDV identified five recombinants among 61 available complete BVDV-1 genomic sequences and confirmed that recombination in BVDV is not rare and can occur among viruses belonging to the same subgenotype or between different subgenotypes [31]. In addition, RNA recombination can occur even between the two species BVDV-1 and BVDV-2 [32]. The existence of recombinant pestiviruses represents a challenge for phylogenetic analysis and classification of virus isolates. In this context, it has been concluded that genotyping of pestivirus isolates should not be based on the analysis of a single genomic fragment [30]. While non-homologous RNA recombination is the major driving force for the generation of various cp virus variants, the existence of a growing number of BVDV subgenotypes is the result of point mutations accumulating over time, also known as genetic drift. In addition, homologous recombination contributes to the genetic diversification of BVDV. The genetic changes can hamper diagnosis of BVDV and may cause failure of protection provided by the established BVDV vaccines [33,34].

## 4. BVDV Variability

Variations among BVDV strains can be evaluated by different methods, including monoclonal antibody reactions, cross-neutralization tests, and a comparison of nucleotide sequences. Phylogenetic analyses of partial and complete genomic sequences provide more detailed information than studies based on reactions with antibodies and allow the rapid detection and discrimination of BVDV-1 and BVDV-2 subgenotypes, as well as the identification of novel subgenotypes. More than two decades ago BVDV isolates were segregated into BVDV-1 and BVDV-2 based on phylogenetic analysis of partial sequences [35,36]. Subsequent studies showed the existence of a growing number of BVDV-1 and BVDV-2 subgenotypes which are described in detail below. Today, it is well known that pestiviruses are genetically highly heterogeneous, even within the individual subgenotypes. 

Different genomic regions, i.e., 5′UTR [7,21,35,36,37,38,39], N^pro^ [3,16,17,35,39,40], E2 [3,16,37,41,42,43,44], NS2-3 [45,46], and NS5B-3′UTR [45,47] have been used for genotyping and classification of BVDV and other pestiviruses. Partial 5′UTR sequences have been most frequently used for phylogenetic analyses and genotyping of BVDV isolates, followed by N^pro^ and E2 coding sequences, and almost all subgenotypes described so far have been classified according to these genomic regions (Table 1, Table 2, Table 3, Table 4 and Table 5). In general, sequence data obtained from one of these genomic regions allow for comparisons with other virus strains only when sequence data of the same genomic region are available. Analyses of complete N^pro^ and E2 coding sequences provide high confidence levels for the allocation of BVDV isolates into established and newly-identified subgenotypes. While the analysis of short partial 5′UTR sequences usually allows correct allocation of virus isolates to the established pestivirus species (e.g., *BVDV-1*) and, in many cases, also to defined subgenotypes, some of the observed BVDV-1 subgenotypes are supported by only low statistical values; consequently, several publications have indicated limitations of inferring BVDV phylogenies using the 5′UTR alone [17,28,30,46]. The main disadvantages of the 5′UTR with regard to its use for phylogenetic analyses are the restricted sequence length and lack of diversity. Consequently, the lack of information does not allow to clearly infer relationships within the major clades, and some branches and BVDV-1 subgenotypes are poorly supported by statistical values. The limited resolution and statistical support observed for phylogenetic analyses of 5′UTR sequences can be significantly improved by analyses of longer sequences and, therefore, it has been recommended to use, e.g., the complete N^pro^ and E2 coding regions, or even the complete polyprotein coding region, for inferring phylogenies and genotyping of pestivirus isolates [17,28].

Due to the lack of standardization and the use of different genomic regions for subgenotyping, inconsistent results have been reported with regard to allocation of some BVDV isolates into subgenotypes [104,162]. For example, the Japanese isolates “IS7NCP/97”, “IS8NCP/97”, and “IS14NCP/99” were placed in the BVDV-1a group according to their 5′UTR regions, but they were placed in the BVDV-1c group when the N^pro^ and E2 coding regions were analyzed [38,45,47]. The same authors reported additional inconsistent results for two other virus isolates. Although Sakoda et al. [167] reported that the “190CP” and “190NCP” isolates belong to the BVDV-1a group according to their 5′UTR sequences, the same isolates were classified in the BVDV-1c group when the same genomic region was analyzed in another study [38,45]. Finally, these isolates were grouped as BVDV-1e when the N^pro^ and E2 coding regions were studied [45,46]. Furthermore, Aguirre et al. [15] described BVDV isolates from llama and alpaca as BVDV-1j using 5′UTR sequences, but based on analysis of E2 coding sequences the same isolates were classified as BVDV-1e. The “So CP/75” isolate was first reported to represent a unique virus belonging to BVDV-1 [38,45] and later classified in the BVDV-1n group by the same authors with another isolate, “Shitara/02/06” [106]. However, their findings conflicted with the results of Xia et al. [46].

Another parameter, which may affect the results of phylogenetic analyses, is the use of various methods, i.e., neighbor-joining, maximum likelihood, or Bayesian methods [46]. While various methods resulted in consistent segregation of BVDV isolates into subgenotypes when the N^pro^ and E2 coding regions were used, analysis of short nucleotide sequences from the 5′UTR can provide conflicting results for some BVDV isolates.

**Figure 1:**
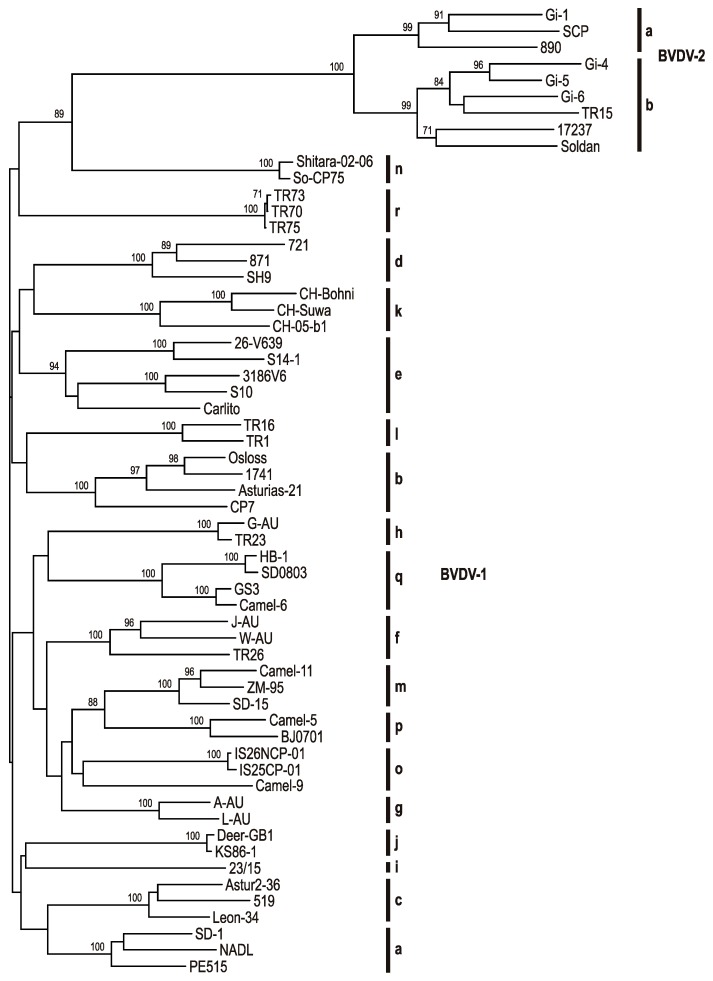
Phylogenetic tree based on full-length N^pro^ encoding sequences of BVDV-1 and BVDV-2 isolates. Phylogenetic analysis of full-length N^pro^ encoding sequences (504 nt) of fifty BVDV-1 and nine BVDV-2 isolates was performed using the neighbor joining method [168,169]. Genetic distances were calculated by the Kimura 2-parameter model [170]. Bootstrap values were calculated for 1000 replicates [171] and are indicated only for statistically significant values (≥70%). The vertical bars and letters indicate the subgenotypes of BVDV-1 (a–r) and BVDV-2 (a and b). GenBank accession numbers of sequence data used for phylogenetic analysis are: Gi-1:AF104030, SCP: U17149, 890:U18059, Gi-4:AF144468, Gi-5:AF144469, Gi-6:AF144470, TR15:EU163979, 17237:EU747875, Soldan:AY735495, Shitara-02-06:AB359930, So-CP75:AB359929, TR73:KF154777, TR70:KF154779, TR75:KF154778, 721:AF144463, 871:AF144462, SH9:AF144473, CH-Bohni:AY894997, Suwa:AY894998, CH-05-b1:EU180037, 26-V639:AF287281, S14-1:AY735490, 3186V6:AF287282, S10:AY735489, Carlito:KP313732, TR16:EU163964, TR1:EU163950, Osloss:M96687, 1741:AF321453, Asturias-21:AY182155, CP7:U63479, G-AU:AF287285, TR23:EU163971, HB-1:KC695812, SD0803:JN400273, GS3:KC695811, 6:KC695810, J:AF287286, W:AF287290, TR26:EU163974, 11:KC207075, ZM-95:AF526381, SD-15:KR866116, 5:KC207071, BJ0701:GU120259, IS26NCP-01:AB359932, IS25CP-01:AB359931, 9:KC207073, A-AU:AF287283, L-AU:AF287287, Deer-GB1:U80902, KS86-1:AB078950, 23-15:AF287279, Astur2-36:AY182162, 519:AF144464, Leon-34:AY182160, SD-1:M96751, NADL:M31182, PE515:EU180034.

After the description of two BVDV-1 subgenotypes in the early 1990s [39], at least twenty-one BVDV-1 subgenotypes (BVDV-1a to -1u) and four BVDV-2 subgenotypes (BVDV-2a to -2d) have been described to date [21,81,49,90,106,121,136,160,161]. The phylogenetic tree based on the N^pro^ coding sequences includes 18 BVDV-1 and two BVDV-2 subgenotypes; for the remaining BVDV-1 and BVDV-2 subgenotypes, complete N^pro^ coding sequences are not available (Figure 1).

According to the literature, a few BVDV isolates could not be allocated into one of the known subgenotypes and, in rare cases, the same subgenotype name was used in different studies for the designation of various BVDV-1 subgenotypes [121,136,160,161]. Apparently, the main reason for this confusing situation is the lack of a harmonized system for segregation of BVDV strains into subgenotypes. Moreover, concurrent submission of articles from different research groups for publication may constitute another reason for duplications in the designation of subgenotypes. 

Currently, at least twenty-one BVDV-1 subgenotypes are either commonly accepted or have been recently suggested. Due to the highly variable structure of pestivirus genomes, it is very likely that additional subgenotypes will be reported. After BVDV-1v to BVDV-1z, we suggest to use two letters or a combination of letters and numbers for the designation of novel subgenotypes. Such a consensus classification system will keep the traditional names used for the established BVDV-1 subgenotypes and will facilitate the segregation of BVDV isolates in the future.

## 5. Global Distribution of BVDV Subgenotypes

Epidemiological studies have shown that various BVDV subgenotypes predominate in different countries. The segregation of BVDV isolates into subgenotypes is shown in Table 1, Table 2, Table 3, Table 4 and Table 5 for the individual continents. Viruses from the established subgenotypes have been detected not only in cattle, but also in pigs and a wide range of ruminant hosts, including sheep, goat, yak, buffalo, llama, alpaca, camel, deer, and bongo [15,16,17,35,42,47,67,80,86,88,103,172].

As noted above, different genomic regions were used for genetic typing of BVDV isolates [3,7,16,17,21,35,36,37,38,39,40,41,42,43,44,100,119,145,160,161] and, hence, it is not possible to create a comprehensive table of BVDV-1 and BVDV-2 subgenotypes on the basis of one single genomic region. Overall, the time periods for sampling vary considerably among the individual studies (summarized in Table 1, Table 2, Table 3, Table 4 and Table 5). Therefore, it was virtually impossible to monitor temporal changes concerning the presence of subgenotypes in various countries. According to the literature, some consecutive studies analyzed the same BVDV isolates and provided consistent results with regard to segregation into subgenotypes. In contrast, for a few other BVDV isolates conflicting results have been reported when different genomic regions were used for genotyping ([38,45,46,105,106], for details see Section 4). Therefore, BVDV isolates with ambiguous segregation to BVDV subgenotypes were excluded from the tables.

With regard to the available published data, 31.6% (2193:6939) of the corresponding BVDV isolates addressed in this study belong to BVDV-1b, while BVDV-1a comprises 20.8% (1443:6939) of the classified isolates, as well as the majority of BVDV vaccine strains. While this analysis of published sequences probably does not reflect the precise distribution of BVDV subgenotypes in individual countries and continents, the calculated percentages provided in the present study can serve as a rough estimate for the presence and frequency of various BVDV subgenotypes. The present collection of data confirms that BVDV-1b is the predominant subgenotype worldwide, followed by BVDV-1a and -1c (Table 6). Considering the individual continents, BVDV-1b is the predominant subgenotype in the Americas, Asia and Europe. In contrast, according to the published data, almost all (95.9%) of the field isolates from Australia have been classified as BVDV-1c (Table 2 and Table 6). Although the total number of analyzed virus isolates from Africa is rather low and not representative for the whole continent, the limited set of data suggests that at least in South Africa BVDV-1a has been detected more frequently than other subgenotypes. The limited number of characterized virus isolates from Africa can be considered as one reason for the lower number of BVDV subgenotypes reported for this continent.

The results of the studies summarized in Table 6 suggest that the worldwide distribution of BVDV-1 including a total of 6117 isolates (88.2%) is significantly broader than the distribution of BVDV-2 isolates, including 822 isolates. The extensive genetic diversity of BVDV reflected by the number of detected subgenotypes has been described for several European countries, as well as for China and Turkey. It can be speculated that this high genetic variability could be related, at least to some extent, to the animal importation policies of these countries [109,173]. In contrast to many European (Table 5) and Asian countries (Table 4), BVDV-1 variation is considerably less developed in the Americas, Australia, and Africa (Table 6). Interestingly, BVDV-1m, -1n, -1o, -1p, and -1q subgenotypes have been detected so far exclusively in Asia. Similarly, the subgenotypes BVDV -1f, -1g, -1h, -1k, -1l, -1r, 1s, and -1t have not been reported to occur in countries outside Europe.

Unfortunately, identical letter codes were used for the designation of different BVDV-1 subgenotypes that were detected and first described at close intervals. After BVDV-1l was used for a newly recognized group of BVDV-1 isolates from Turkey in 2008 [160], the same subgenotype name was used for another distinct group of virus isolates reported in the same year [121]. Additionally, a similar conflict concerns the recently added subgenotype BVDV-1r which was initially used for the description of a distinct group of BVDV-1 isolates from Turkey in 2014 [161], but later the same designation was used for a group of different BVDV-1 isolates from Italy that was first described in 2015 [136]. 

BVDV-2 was first identified in Canada and the United States and the high prevalence reported in the 1990s did not significantly change during the past twenty years. Analyses over the past two decades showed the presence of BVDV-2 in a number of European countries, including Germany, Belgium, France, the United Kingdom, Slovakia, and Austria [174]. Further studies revealed an even broader distribution of BVDV-2, including virus isolates from all inhabited continents. BVDV-2a is the most prevalent subgenotype of BVDV-2 on all continents. BVDV-2c has been detected only in Europe and the Americas. One single contaminating BVDV strain from Argentina was classified as BVDV-2d, but additional members of this suggested subgenotype have not been detected since it was reported in 1995 [49].

## 6. Conclusions

Phylogenetic analyses of BVDV isolates can provide useful insights into the genetic relatedness among these viruses, which are either endemically present in an area for a longer time period or have been recently introduced, e.g., by animal imports. Accordingly, studies on molecular epidemiology of BVDV can assist in tracing virus isolates circulating in individual countries and globally. To date at least twenty-five different BVDV-1 and BVDV-2 subgenotypes have been described. However, a standardized and commonly-accepted system for genetic typing of BVDV isolates has not been established so far. Developing internationally-harmonized rules for discrimination and designation of BVDV subgenotypes will help to reduce inconsistencies in molecular typing of BVDV. Detailed knowledge about the variability of BVDV also provides useful information for evaluating the success of disease control programs. Moreover, different levels of cross-protection between highly variable BVDV-1 and BVDV-2 subgenotypes might be implicated in the success of vaccination programs.

## Figures and Tables

**Table 1:** Distribution of Bovine viral diarrhea virus (BVDV) subgenotypes in the Americas.

Country	Genomic Region	Year of Isolation	BVDV-1																						BVDV-2					Reference
			a	b	c	d	e	f	g	h	i	j	k	l	m	n	o	p	q	r	s	t	u	?	a	b	c	d	?	
Argentina	5′UTR, N^pro^, E2	1984–2010	23	36	-	-	-	-	-	-	-	-	-	-	-	-	-	-	-	-	-	-	-	2	5	4	1	1	4	(I)
Brazil	5′UTR, N^pro^, E2	1994–2016	54	20	4	24	1	-	-	-	1	-	-	-	-	-	-	-	-	-	-	-	-	2	2	50	-	-	3	(II)
Peru and Chile	5′UTR	1993–2004	3	29	2	-	-	-	-	-	-	-	-	-	-	-	-	-	-	-	-	-	-	-	-	-	-	-	5	(III)
USA	5′UTR, N^pro^, E2	1971–2015	184	652	-	-	-	-	-	-	-	-	-	-	-	-	-	-	-	-	-	-	-	3	129	4	-	-	125	(IV)
Canada	5′UTR	1990–1993	1	1	-	-	-	-	-	-	-	-	-	-	-	-	-	-	-	-	-	-	-	-	3	-	-	-	-	(V)
Uruguay	5′UTR, N^pro^	2014	12	-	-	-	-	-	-	-	1	-	-	-	-	-	-	-	-	-	-	-	-	-	-	1	-	-	-	(VI)
**Total number**			**277**	**738**	**6**	**24**	**1**				**2**													**7**	**139**	**59**	**1**	**1**	**137**	

?: Genotyping was not performed. References (I): [48,49,50,51,52]; (II): [7,43,49,52,53,54,55,56,57,58]; (III): [59,60]; (IV): [39,44,49,61,62,63,64,65,66,67,68,69,70]; (V): [49,71]; (VI): [72]. In [57] the year of virus isolation was not displayed in the study. 5′UTR: 5′ untranslated regions.

**Table 2:** Distribution of BVDV subgenotypes in Australia.

Country	Genomic Region	Year of Isolation	BVDV-1																					BVDV-2				Reference
			a	b	c	d	e	f	g	h	i	j	k	l	m	n	o	p	q	r	s	t	u	a	b	c	d	
Australia	5′UTR, N^pro^	1971–2005	13	1	425	-	-	-	-	-	-	-	-	-	-	-	-	-	-	-	-	-	-	4	-	-	-	(I)
**Total number**			**13**	**1**	**425**																			**4**				

References (I): [39,73].

**Table 3:** Distribution of BVDV subgenotypes in Africa.

Country	Genomic Region	Year of Isolation	BVDV-1																						BVDV-2					Reference
			a	b	c	d	e	f	g	h	i	j	k	l	m	n	o	p	q	r	s	t	u	?	a	b	c	d	?	
Egypt	5′UTR, N^pro^	1994–2004	-	4	-	-	-	-	-	-	-	1	-	-	-	-	-	-	-	-	-	-	-	-	-	-	-	-	-	(I)
Tunisia	5′UTR, N^pro^	2001–2002	-	2	-	-	-	-	-	-	-	-	-	-	-	-	-	-	-	-	-	-	-	-	3	-	-	-	-	(II)
South Africa	5′UTR	1990–2009	31	13	20	20	-	-	-	-	-	-	-	-	-	-	-	-	-	-	-	-	-	20	-	-	-	-	-	(III)
**Total number**			**31**	**19**	**20**	**20**						**1**												**20**	**3**					

References (I): [52,74,75]; (II): [76]; (III): [77,78,79].

**Table 4:** Distribution of BVDV subgenotypes in Asia.

Country	Genomic Region	Year of Isolation	BVDV-1																						BVDV-2					Reference
			a	b	c	d	e	f	g	h	i	j	k	l	m	n	o	p	q	r	s	t	u	?	a	b	c	d	?	
China	5′UTR, N^pro^, E2	2005–2013	15	113	17	13	-	-	-	-	-	-	-	-	116	-	5	9	14	-	-	-	22	10	2	1	-	-	12	(I)
India	5′UTR, N^pro^, E^rns^-E1, E2, NS5B	2000–2010	-	23	6	-	-	-	-	-	-	-	-	-	-	-	-	-	-	-	-	-	-	-	3	1	-	-	-	(II)
Philippine	E2	-	-	3	-	-	-	-	-	-	-	-	-	-	-	-	-	-	-	-	-	-	-	-	-	-	-	-	-	(III)
Japan	5′UTR, N^pro^	1975–2006	216	558	226	-	-	-	-	-	-	4	-	-	1	2	2	-	-	-	-	-	-	2	315	-	-	-	2	(IV)
Korea	5′UTR	2005–2015	21	6	2	-	-	-	-	-	-	-	-	-	-	1	-	-	-	-	-	-	-	-	18	-	-	-	1	(V)
Mongolia	5′UTR	2014	4	-	-	-	-	-	-	-	-	-	-	-	-	-	-	-	-	-	-	-	-	-	4	-	-	-	-	(VI)
**Total number**			**256**	**703**	**251**	**13**						**4**			**117**	**3**	**7**	**9**	**14**				**22**	**12**	**342**	**2**			**15**	

?: genotyping was not performed. References (I): [80,81,82,83,84,85,86,87,88,89,90,91,92,93,94,95,96,97,98,99]; (II): [47,100,101,102,103,104]; (III): [49]; (IV): [37,49,105,106,107,108,109]; (V): [110,111,112]; (VI): [113]. In [49], [83], [85] and [89] the year of virus isolation was not displayed in the study.

**Table 5:** Distribution of BVDV subgenotypes in Europe.

Country	Genomic Region	Year of Isolation	BVDV-1																						BVDV-2					Reference
			a	b	c	d	e	f	g	h	i	j	k	l	m	n	o	p	q	r	s	t	u	?	a	b	c	d	?	
Austria	5′UTR, N^pro^	1997–2006	4	52	-	33	6	142	7	154	-	-	3	-	-	-	-	-	-	-	-	-	-	1	1	-	-	-	3	(I)
Belgium	E2	1991–2002	1	19	-	-	-	-	-	-	-	-	-	-	-	-	-	-	-	-	-	-	-	4	6	-	-	-	7	(II)
Croatia	5′UTR, N^pro^	2007–2011	-	11	-	-	-	7	-	-	-	-	-	-	-	-	-	-	-	-	-	-	-	-	-	-	-	-	-	(III)
Czech Republic	5′UTR, N^pro^	2004–2007	-	16	-	16	2	7	-	-	-	-	-	-	-	-	-	-	-	-	-	-	-	-	-	-	-	-	-	(IV)
Denmark	5′UTR, E2	1962–2012	-	16	-	32	1	-	-	-	-	-	-	-	-	-	-	-	-	-	-	-	-	-	-	-	-	-	-	(V)
Finland	5′UTR, N^pro^	1994–2004	-	-	-	5	-	1	-	-	-	2	-	-	-	-	-	-	-	-	-	-	-	-	-	-	-	-	-	(VI)
France	5′UTR, N^pro^	1993–2005	3	15	-	3	46	-	-	-	-	-	-	3^▲^	-	-	-	-	-	-	-	-	-	-	2	-	-	-	3	(VII)
Germany	5′UTR, E2	1960–2014	1	31	-	24	24	65	3	17	-	-	1	-	-	-	-	-	-	-	-	-	-	-	11	-	16	-	-	(VIII)
Hungary	5′UTR, N^pro^	1971–1998	-	2	-	-	-	3	-	-	-	-	-	-	-	-	-	-	-	-	-	-	-	-	-	-	-	-	-	(IX)
Ireland	5′UTR	1968–2014	428	19	-	1	1	-	-	-	-	-	-	-	-	-	-	-	-	-	-	-	-	-	-	-	12	-	-	(X)
Italy	5′UTR, N^pro^	1966–2016	16	193	2	27	141	55	8	20	-	-	3	1^▲^	-	-	-	-	-	2^▲^	1	1	2	8	10	-	-	-	5	(XI)
Kosovo	5′UTR	2011	-	3	-	-	-	-	-	-	-	-	-	-	-	-	-	-	-	-	-	-	-	-	-	-	-	-	-	(XII)
Poland	5′UTR, N^pro^	2004–2011	-	31	-	24	-	8	2	-	-	-	-	-	-	-	-	-	-	-	-	-	-	-	4	-	-	-	-	(XIII)
Portugal	5′UTR	-	6	19	-	3	3	-	-	-	-	-	-	-	-	-	-	-	-	-	-	-	-	-	1	2	-	-	-	(XIV)
Slovakia	5′UTR, N^pro^	1994–2004	-	-	-	1	1	1	-	1	-	-	-	-	-	-	-	-	-	-	-	-	-	-	1	-	-	-	-	(XV)
Slovenia	5′UTR, N^pro^, C	1997–2006	-	4	-	17	1	21	1	-	-	-	-	-	-	-	-	-	-	-	-	-	-	-	-	-	-	-	-	(XVI)
Spain	5′UTR, N^pro^	1989–2015	3	162	2	9	8	2	-	2	-	-	1	1	-	-	-	-	-	-	-	-	-	-	2	6	-	-	-	(XVII)
Sweden	5′UTR, N^pro^	2002–2004	7	28	-	77	-	-	-		-	-	-	-	-	-	-	-	-	-	-	-	-	-	-	-	-	-	-	(XVIII)
Switzerland	5′UTR, N^pro^	2008–2012	-	35	-	-	137	1	-	114	-	-	71	-	-	-	-	-	-	-	-	-	-	1	-	-	-	-	-	(XIX)
Turkey	5′UTR, N^pro^, C, E^rns^, E2	1997–2012	7	11	-	7	-	20	-	1	1	-	-	34^▲^	-	-	-	-	-	3^▲^	-	-	-	7	5	1	-	-	14	(XX)
United Kingdom	5′UTR, N^pro^	1966–2011	390	65	-	2	5	1	-	-	23	1	-	-	-	-	-	-	-	-	-	-	-	3	7	-	-	-	-	(XXI)
**Total number**			**866**	**732**	**4**	**281**	**376**	**334**	**21**	**309**	**24**	**3**	**79**	**39**						**5**	**1**	**1**	**2**	**24**	**50**	**9**	**28**		**32**	

?: genotyping was not performed. ^▲^: Isolates from different clusters, but given the same name. References (I): [21,49,114,115,116]; (II): [41,49]; (III): [117]; (IV): [118]; (V): [119,120]; (VI): [52]; (VII): [21,49,121]; (VIII): [122,123,124,125,126]; (IX): [21]; (X): [127,128,129]; (XI): [21,49,130,131,132,133,134,135,136,137,138,139,140]; (XII): [141]; (XIII): [142,143]; (XIV): [144]; (XV): [21,49,52]; (XVI): [145,146]; (XVII): [21,40,147,148,149,150,151,152]; (XVIII): [153]; (XIX): [9,154,155,156]; (XX): [157,158,159,160,161,162]; (XXI): [21,49,163,164,165,166]. In [9], the complete values were closest to the integral numbers, while the proportional values given in the related study are transformed into numerical data. In [125], certain number of the isolates segregated into subgenotypes 1b, 1d, 2a, and 2c are not indicated. In [144] and [166], the year of virus isolation was not displayed in the study. In [162] and [163] different typing regions were used. Samples are included into the unknown subgenotype category in case of ambiguous typing results reported for different regions.

**Table 6:** Continental distribution of BVDV subgenotypes *.

Country	Genomic Region	Year of Isolation	BVDV-1																						BVDV-2				
			a	b	c	d	e	f	g	h	i	j	k	l	m	n	o	p	q	r	s	t	u	?	a	b	c	d	?
Americas	5′UTR, N^pro^, E2	1971–2010	277	738	6	24	1	-	-	-	2	-	-	-	-	-	-	-	-	-	-	-	-	7	139	59	1	1	137
Australia	5′UTR, N^pro^	1971–2005	13	1	425	-	-	-	-	-	-	-	-	-	-	-	-	-	-	-	-	-	-	-	4	-	-	-	-
Africa	5′UTR, N^pro^	1994–2004	31	19	20	20	-	-	-	-	-	1	-	-	-	-	-	-	-	-	-	-	-	20	3	-	-	-	-
Asia	5′UTR, N^pro^, E^rns^-E1, E2, NS5B	1975–2012	256	703	251	13	-	-	-	-	-	4	-	-	117	3	7	9	14		-	-	22	12	342	2	-	-	15
Europe	5′UTR, N^pro^, C, E^rns^, E2	1962–2012	866	732	4	281	376	334	21	309	24	3	79	39	-	-	-	-	-	5	1	1	2	24	50	9	28	-	32
**Total number/individual subgenotype**			1443	2193	706	338	377	334	21	309	26	8	79	39	117	3	7	9	14	5	1	1	24	63	538	70	29	1	184
**Total number/species**			6117																						822				

* In order to avoid duplications, isolates from some consecutive studies which evaluated the isolates without identification were not included in the tables. ?: genotyping was not performed.
